# Managing the Costs of Routine Follow-up Care After Living Kidney Donation: a Review and Survey of Contemporary Experience, Practices, and Challenges

**DOI:** 10.1007/s40472-022-00379-w

**Published:** 2022-09-22

**Authors:** Krista L. Lentine, Nagaraju Sarabu, Gwen McNatt, Robert Howey, Rebecca Hays, Christie P. Thomas, Ursula Lebron-Banks, Linda Ohler, Cody Wooley, Addie Wisniewski, Huiling Xiao, Andrea Tietjen

**Affiliations:** 1 Saint Louis University Transplant Center, Saint Louis University Center for Abdominal Transplantation, 1201 S. Grand Blvd, St. Louis, MO 63104 USA; 2 University Hospitals Cleveland Medical, Cleveland, OH USA; 3 University of Iowa Carver College of Medicine, Iowa City, IA USA; 4 Toyon Associates, Inc., Concord, CA USA; 5 University of Wisconsin Hospital and Clinics, Madison, WI USA; 6 New York-Presbyterian Hospital, New York, NY USA; 7 New York University, New York, NY USA; 8 Saint Barnabas Medical Center, Livingston, NJ USA

**Keywords:** Billing, Follow-up care, Living kidney donors, Reimbursement, Surveillance

## Abstract

**Purpose of Review:**

While living organ donor follow-up is mandated for 2 years in the USA, formal guidance on recovering associated costs of follow-up care is lacking. In this review, we discuss current billing practices of transplant programs for living kidney donor follow-up, and propose future directions for managing follow-up costs and supporting cost neutrality in donor care.

**Recent Findings:**

Living donors may incur costs and financial risks in the donation process, including travel, lost time from work, and dependent care. In addition, adherence to the Organ Procurement and Transplantation Network (OPTN) mandate for US transplant programs to submit 6-, 12-, and 24-month postdonation follow-up data to the national registry may incur out-of-pocket medical costs for donors. Notably, the Centers for Medicare and Medicaid Services (CMS) has explicitly disallowed transplant programs to bill routine, mandated follow-up costs to the organ acquisition cost center or to the recipient’s Medicare insurance. We conducted a survey of transplant staff in the USA (distributed October 22, 2020–March 15, 2021), which identified that the mechanisms for recovering or covering the costs of mandated routine postdonation follow-up at responding programs commonly include billing recipients’ private insurance (40%), while 41% bill recipients’ Medicare insurance. Many programs reported utilizing institutional allowancing (up to 50%), and some programs billed the organ acquisition cost center (25%). A small percentage (11%) reported billing donors or donors’ insurance.

**Summary:**

To maintain a high level of adherence to living donor follow-up without financially burdening donors, up-to-date resources are needed on handling routine donor follow-up costs in ways that are policy-compliant and effective for donors and programs. Development of a government-supported national living donor follow-up registry like the Living Donor Collective may provide solutions for aspects of postdonation follow-up, but requires transplant program commitment to register donors and donor candidates as well as donor engagement with follow-up outreach contacts after donation.

**Supplementary Information:**

The online version contains supplementary material available at 10.1007/s40472-022-00379-w.

## Introduction

Living donor kidney transplantation not only offers the best treatment option to patients in need of renal replacement therapy but also provides cost-savings to the healthcare system, compared to both dialysis and deceased donor transplantation. A contemporary discrete event model analysis of a hypothetical cohort of 20,000 end-stage renal disease patients found that almost all living donor kidney transplants result in cost savings, ranging from $13,000 USD to $30,000 USD, and even higher risk transplants from HLA-incompatible donors were cost-effective [1]. In the USA, in 2014, the American Society of Transplantation (AST) Living Donor Community of Practice (LDCOP) held a consensus conference to identify best practices, knowledge gaps, and systemic barriers in living kidney donation [2]. Recognizing that donation benefits recipients and society, attendees endorsed that living donation should be a financially neutral act [3]. Moreover, the consensus report recommended the provision of standardized guidance for billing processes to reduce donor financial burdens, which lead to the formation of an AST LDCOP Financial Workgroup [3]. Concerted approaches to understanding and overcoming financial risks for living organ donors, including education and informed consent, research to better quantify financial risks, and policies to mitigate such risks remain an important priority to preserve the economic health of living donors [4].

The follow-up of living organ donors has been a topic of long-standing controversy in the field of organ donation [5], in part, because of debate around responsibility for the associated costs, and whether there is sufficient need to warrant the effort and costs. The health risks of living donation are generally accepted as sufficiently low to justify the practice, but short- and long-term risks have been identified [6–8]. Notably, much of the evidence to define risks have been limited by short observation periods, a high proportion of loss to follow-up, insufficient power to quantify rare events, and limited racial and ethnic diversity of study cohorts [9–11]. To foster collaboration between transplant programs and donors and to help ensure that commitment to donor care extends to health beyond the donation event, in 1999, the Organ Procurement and Transplantation Network (OPTN) started requesting transplant centers in the USA to submit living donor follow-up forms at 6-month and 12-month postdonation [12]. Additional elements such as insurance status were added in 2004 and the duration of follow-up was extended to 24 months in 2008 (Table 1). In 2013, the OPTN defined minimum transplant center-level follow-up thresholds as at least 80% and 70% for clinical and laboratory data, respectively [13]. Two decades later, postdonation follow-up remains a challenge, especially for vulnerable groups like uninsured donors [14].

**Table 1:** Organ Procurement and Transplantation Network (OPTN)-mandated data elements for transplant centers to collect and report on living kidney donors at 6-, 12-, and 24-months postdonation

**Donor status and clinical information (all are required)**
1. Patient status (alive, deceased)
2. Working for income, and if not working, reason for not working
3. Loss of medical (health, life) insurance due to donation
4. Has the donor been readmitted since last LDR or LDF form was submitted?
5. Kidney complications
6. Regularly administered dialysis as an ESRD patient
7. Hypertension requiring medication
8. Diabetes
9. Cause of death, if applicable and known
**Kidney laboratory data (all are required)**
1. Serum creatinine
2. Urine protein

Adapted from Organ Procurement and Transplantation Network policy for data requirements for living kidney donor, policy 18.5 [44]. *LDR*, Living Donor Registration; *LDF*, Living Donor Follow-up

In this article, we review current understanding of the financial risks associated with living kidney donation, summarize the evolution of OPTN mandated living donor follow-up, describe how transplant programs manage the costs of routine living kidney donor (LKD) follow-up in contemporary practice, and propose future directions to improve financial neutrality for donors. This manuscript is a work product of the AST LDCOP.

## Financial Risks of Living Kidney Donation and the Intersection with Follow-up Costs

While living donor kidney transplantation is cost-saving for the healthcare system, it is notable that living donors incur costs in the donation process. In a recent systematic review of publications on donation-related expenses, Fu et al., found that LKDs incurred direct costs ranging between $900 USD to $19,900 USD in the period starting from evaluation to 1-year postdonation [15]. In addition, indirect costs such as lost wages for donors and caregivers were also identified. The economic impacts of donation may extend beyond direct costs to longer-term impacts on employment and socioeconomic status [16]. A survey of 51 U.S. donors found that perceived financial burden was the highest among LKDs with predonation cost concerns and low income [17]. A single-center, retrospective survey of LKDs who were employed at the time of donation (2005–2015) found that longer time-off from work was a significant predictor of financial burden, and that donors in manual/skilled trade occupations were particularly vulnerable [18]. Older age at donation and nondirected (vs. directed) donation were associated with a significantly decreased financial burden [18]. Living kidney donation may also lead to challenges in obtaining life, disability, and health insurance [19–21].

While the goal of the OPTN follow-up mandate is to support the medical safety of donation, it should be noted that currently, there is no formal policy for donors or programs to recuperate the costs of complying with this follow-up mandate. Practice variation across programs in terms of interpretation and use of the Centers for Medicare and Medicaid Services (CMS) cost report, and in access to other resources, can result in significant costs passed on to donors [22, 23]. Completing the required follow-up tests at a primary care provider's office, rather than at the transplant center, is policy-compliant but may result in LKDs’ incurring costs, and requires transplant center staff time to collect the data. Further, to comprehensively capture the impact of donation, it has been recommended that the scope of long-term follow-up extend beyond solely medical outcomes to psychosocial and economic outcomes [24].

## Contemporary Billing Practices for Living Kidney Donor Follow-up Care Costs: a Survey of US Transplant Programs

Although direct costs associated with OPTN-mandated postdonation medical follow-up are small compared to costs associated with living donor evaluation and surgery, managing these costs poses financial challenges for transplant programs and donors. Notably, CMS has explicitly disallowed billing donor follow-up costs to the Organ Acquisition Cost Center (OACC) on the Medicare Cost Report (MCR) or to a recipient’s Medicare [25].

To help inform the community of current practices and guide discussions of effective strategies for managing follow-up costs, the AST LDCOP Financial Workgroup developed and conducted a contemporary survey of staff at US LKD programs on practices in handling living donor follow-up costs (Table S1). The survey was administered to staff at US LKD programs (administrators, nephrologists, surgeons, coordinators, and social workers). Data were analyzed from distribution October 22, 2020 to March 15, 2021. The first page of the survey noted that the decision to proceed indicates consent to participate and that responses are reported anonymously. All human subject procedures complied with all applicable ethical standards (including the Helsinki declaration and its amendments, institutional/national research committee standards, and international/national/institutional guidelines). This survey study was approved by the Saint Louis University Institutional Review Board (IRB protocol #31,418).

Each transplant program was represented once in the primary analysis. Representative responses from programs with multiple respondents were selected using a hierarchical algorithm, similar to previous methods [26–30]. For the current survey, we prioritized responses from administrators, followed by surgeons or nephrologists, then coordinators. Finally, if there was still more than one response per transplant center, we retained the most recent (latest) response. We received 156 survey responses from staff at US LKD programs; 56 responses were from a center with one survey respondent and 100 responses were from programs with more than one respondent. After limiting to unique program responses, 93 program responses were available for primary analyses, representing 48% of U.S. living donor recovery programs in the study period. Respondents practiced in 35 states, and all United Network for Organ Sharing (UNOS) regions were represented.

To assess possible relationships between program volume, follow-up rates, and billing practices, we examined Scientific Registry of Transplant Recipients (SRTR) data. For the representation of living donor recovery volume, we considered a period of 12 months before the beginning of the COVID-19 pandemic (March 2019–February 2020). For baseline follow-up rates, we considered complete 6-month follow-up rates (clinical and laboratory testing) for living donations from October 2018 to September 2019, to allow time for reporting before the pandemic. Twenty-five programs were categorized as “smaller” and 68 as “larger” volume based on the median (*n* = 18) of all active transplant programs; 49 were classified as having “lower” and 44 as having “higher” rates of follow-up, based on the median of 6-month follow-up compliance (83%).

### Handling the Costs of Follow-up Care

**Fig. 1:**
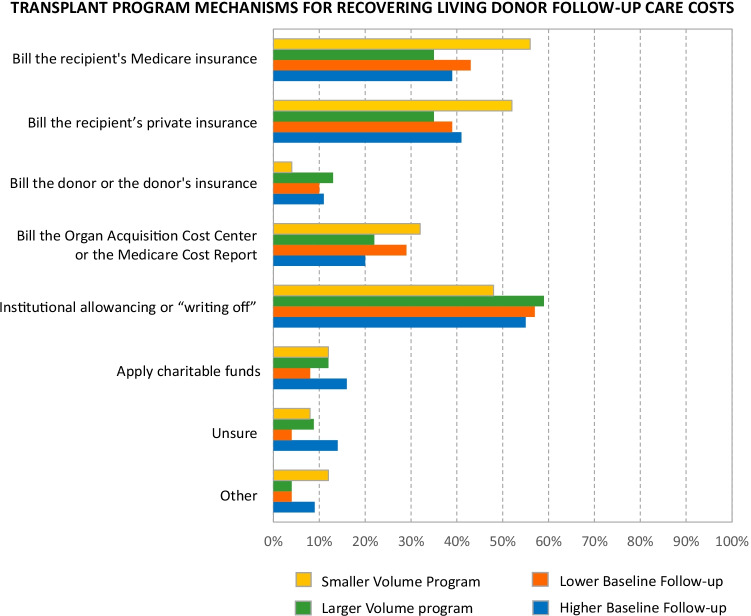
Mechanisms for recovering living kidney donor (LKD) follow-up care costs: results from a US transplant program survey distributed from October 22, 2020 to March 15, 2021 (*N* = 93). *A total of 93 transplant programs responded. Transplant programs were categorized as “smaller” (*n* = 25) and “larger” (*n* = 68) volume group based on whether annual LKD volume was below or above the median, and as “lower” (*n* = 49) or “higher”(*n* = 44) follow-up rate group, based on 6-month follow-up above or below the median

Most survey respondents (82%) reported having standardized policies and procedures for handling living donor follow-up costs, which was true regardless of volume (80% of “smaller” volume centers vs. 82% of “larger” volume centers). Nearly one in five program respondents (17%) reported wanting clarity on cost management. The most commonly reported mechanism for recovering follow-up care costs was billing the recipient’s insurance, and notably, billing the recipient’s Medicare was as common as billing private insurance (41% vs. 40%). Examined by transplant program volume, billing the recipient’s Medicare was more common at smaller compared to larger volume programs (56% vs. 35%). A similar pattern was observed for billing the recipient’s private insurance (52% vs. 35%). Many programs reported utilizing institutional allowancing, which was reported more by larger compared to smaller volume programs (59% vs. 48%). Some programs reported accumulating follow-up costs on their OACC and then recording them on the MCR (25%). The practice of using the MCR tended to be more common at smaller than at larger volume programs (32% vs. 22%), and at programs with lower vs. higher follow-up performance (29% vs. 20%). Eleven percent of programs reported billing donors or donor insurance. Some reported use of charitable funds (12%), which was more common at centers with higher vs. lower follow-up performance (16% vs. 8%) (Table 2, Fig. 1).

**Table 2:** Reported mechanisms and practices for covering or recovering the costs of routine LKD follow-up care: results from a US transplant program survey distributed from October 22, 2020 to March 15, 2021 (*n* = 93)

Please check all that apply with respect to managing the costs of OPTN-mandated, routine postdonation living kidney donor follow-up at your transplant program	% (*n*)
Our program has a standardized policy and procedure on how to handle these costs	82% (76)
Our program needs clarity on the provision of these costs	17% (16)
Other	4% (4)
What mechanisms does your program use for covering or recovering the costs of OPTN-mandated, routine postdonation living kidney donor follow-up?	
	% (*n*)
Bill the recipient’s Medicare insurance	41% (38)
Bill the recipient’s private insurance	40% (37)
Bill the donor or the donor’s insurance	11% (10)
Bill the organ acquisition or the Medicare cost report	25% (23)
Institutional allowancing or “writing off” costs	56% (52)
Apply charitable funds	12% (11)
Unsure	9% (8)
Other	7% (6)

For these “select all that apply” questions, column total percentage may exceed 100% because each respondent was permitted to select more than one option. Free-text comments were grouped into thematic categories (Table S2)

### Donor Candidate Education Regarding the Costs of Follow-up Care

**Fig. 2:**
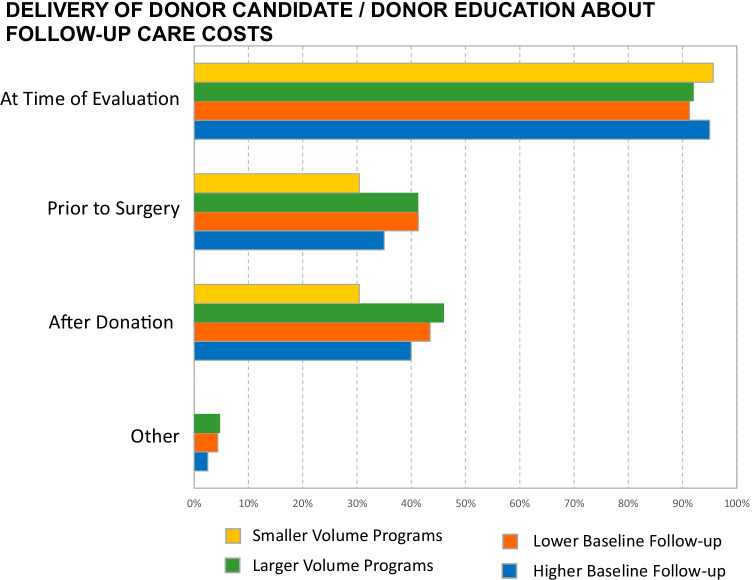
Delivery of living kidney donor (LKD) candidate / living donor education about follow-up care costs (*N* = 93). Transplant programs were categorized as “smaller” (*n* = 25) and “larger” (*n* = 68) volume group based on whether annual LKD volume was below or above the median, and as “lower” (*n* = 49) or “higher”(*n* = 44) follow-up rate group, based on 6-month follow-up above or below the median

Most responding programs reported educating donor candidates on costs and coverage of routine follow-up (89%) but a minority reported they do not (11%). Education practices did not differ by either program volume or by rates of baseline follow-up. Donor education most commonly occurred at the time of evaluation (93%), while under one-half of programs educated at the time of follow-up (42%). Some programs also educated donors about follow-up care costs before surgery (38%) (Table 3, Fig. 2). Themes from free-text responses were consistent with the primary survey response selections (Table S2).

**Table 3:** Education of living kidney donors on the costs of follow-up care: results from a US transplant program survey distributed from October 22, 2020 to March 15, 2021

Does your program educate living donor candidates / donors on the costs and coverage for OPTN-mandated postdonation routine follow-up care? (*N* = 93)	% (*n*)
Yes	89% (83)
No	11% (10)
If your program educates living donor candidates / donors on the costs and coverage for OPTN-mandated postdonation routine follow-up care, when does this education occur? (*N* = 83)	% (*n*)
At time of evaluation	93% (77)
Prior to surgery	38% (32)
After donation, when follow-up must be conducted	42% (35)
Other	4% (3)

For these “select all that apply” questions, column total percentage may exceed 100% because each respondent was permitted to select more than one option. Free-text comments were grouped into thematic categories (Table S2)

## Managing Postdonation Follow-up: the Need for Ongoing Support and Education

The 2013 OPTN living donor follow-up policy was not accompanied by specific guidance on how to recover or manage associated costs. Our survey reveals a range of mechanisms being employed, most commonly including billing recipient insurance (Medicare or private plan), institutional allowancing, and billing the MCR. These contemporary practice patterns differ from those identified in a 2007–2008 survey when billing donor insurance was the most common method [31]. In two surveys of transplant centers addressing living donor follow-up practices conducted before the 2013 OPTN mandate, respondents endorsed a lack of reimbursement mechanisms as one of the most important barriers to compliance with donor follow-up requirements [31, 32]. One of these earlier surveys that reported donor insurance was the most commonly utilized mechanism for cost recovery was limited by small sample size, and only 36 responses were received for that question [31]. Since then, a few programs have described positive outcomes associated with designating funds to cover donor follow-up [33].

Some transplant programs may have misinterpreted the CMS manual and sought to recover LKD follow-up care costs via the MCR or recipient Medicare billing to fulfill the OPTN mandate and recover associated costs. Billing the recipient’s Medicare and using the MCR tend to occur more at smaller compared to larger volume programs, and at centers with lower vs. higher baseline follow-up performance. Thus, broader-reaching education for transplant programs is needed on billing practices that are compliant with the CMS manual. We recently outlined appropriate mechanisms for recovery of follow-up care costs [34], which include (1) billing recipient non-Medicare insurance unless excluded in the insurer's contract, (2) covering costs with institutional or charitable funds [33, 35], (3) allowancing or writing-off at the transplant center level, and (4) billing donors or donor insurance (for services either at the transplant program or at a primary care physician’s office).

## Future Directions for Managing Living Donor Follow-up Costs

Living donor candidates must be counseled in detail about the potential financial consequences of donation during the evaluation. Failing to counsel appropriately on financial risks may compromise the ability of donor candidates to provide informed consent. Living donors should also be educated on and assisted in accessing reimbursement programs for donation-associated costs, such as the US federally-funded Living Donor Assistance Center (NDLAC), and the National Kidney Registry’s “Living Donor Shield” when applicable [36, 37]. We also contend that universal access to living donor follow-up is important in the first few years to monitor health and to instill in donors the value of engaging in long-term routine screening. As such, there is a rationale to include living donor follow-up care as a covered benefit under OACC. Uniform access to follow-up care is especially important in the USA, where there are significant disparities in access to healthcare and living donor outcomes based on social determinants of health and other factors [14]. Provision of donor follow-up care may also reduce financial burdens on donors, and provide early warning systems for donors at increased risk of long-term complications [38].

We also support the concept of national tracking of donor outcomes and posit that gaps in funding mechanisms may limit such efforts in the USA. Successful models to acquire information on living donor outcomes exist elsewhere, particularly in countries with socialized medicine. For example, all living donors in Switzerland are registered in the Swiss Organ Living Donor Health Registry, which collects information from general practitioners at 1-year after donation and biennially thereafter [39]. Australia and New Zealand have a similar universal system [40]. In the USA, the Living Donor Collective is a Health Services Research Administration (HRSA)-supported initiative of the SRTR to create a lifetime registry for all donor candidates evaluated at US transplant centers [41, 42], for which pilot phase experience (June 2018–September 2020) was recently published [43]. Under the Living Donor Collective model, centers register donor candidates, and the SRTR is responsible for follow-up. Development of a government-supported national living donor follow-up registry like the Living Donor Collective may provide solutions for aspects of postdonation follow-up, but requires transplant program commitment to register donors and donor candidates, as well as donor engagement with follow-up outreach contacts after donation.

## Conclusion

A path towards mitigating health and financial risks of living kidney donation includes maintaining a high level of adherence to complete living donor follow-up without financially burdening donors. We advocate for the revision of OACC policy to include follow-up costs as part of the commitment necessary for living donor care and safety, rather than solely for data collection. The SRTR Living Donor Collective may also provide a solution for long-term follow-up [41, 42], but requires a partnership of transplant centers in registering donors and donor engagement to be successful. Ongoing efforts to support follow-up for all living donors, including attention to covering necessary costs and removing financial barriers, are vital to help ensure opportunities for safe donation, especially among vulnerable groups.

## Supplementary Information

Below is the link to the electronic supplementary material.

Supplementary file1 (PDF 445 KB)

## Data Availability

Data availability is limited to aggregate summaries as reported, based on IRB requirements.
