# Supplementary material for: Managing the Costs of Routine Follow-up Care After Living Kidney Donation: a Review and Survey of Contemporary Experience, Practices, and Challenges
Source: Curr Transplant Rep. 2022 Sep 22;9(4):328–35. doi: 10.1007/s40472-022-00379-w (PMC9510404; doi:10.1007/s40472-022-00379-w)
Supplement: Supplementary file 1 — Supplementary file1 (PDF 445 KB) [file 40472_2022_379_MOESM1_ESM.pdf]

## SUPPLENTARY APPENDIX

**Table S1. Survey Instrument**

| **Survey Questions** | **Response Format** | **Response Options** |
|---|---|---|
| What is your role at your transplant center? | Single Answer | • Administrator<br>• Financial Coordinator<br>• Clinical Coordinator<br>• Social Worker<br>• Surgeon<br>• Nephrologist<br>• Other |
| What is your center's 4-character UNOS code? | Dropdown Menu | • UNOS Code |
| Please check all that apply with respect to the costs and coverage for OPTN/UNOS mandated post donation living donor routine follow-up | Check all that apply | • Our program has a standardized policy and procedure on how to handle these costs<br>• Our program needs clarity on the provision of these costs<br>• Other, specify: |
| What mechanisms does your program use for recovering or covering the costs of OPTN/UNOS mandated post donation living donor routine follow-up? | Check all that apply | • Bill the recipient's Medicare insurance<br>• Bill the recipient's private insurance<br>• Bill the donor or the donor's insurance<br>• Bill to the Organ Acquisition or the Medicare Cost Report<br>• Institutional allowancing or writing off costs<br>• Apply charitable funds<br>• Unsure<br>• Other, specify: |
| Does your program educate donor candidate on the costs and coverage for OPTN/UNOS mandated post donation living donor routine follow-up? | Categorical | • Yes<br>• No |
| If your program educates your donor on the costs and coverage for OPTN/UNOS mandated post donation living donor routine follow-up, when does this education occur? | Check all that apply | • At time of evaluation<br>• Prior to surgery<br>• After donation, when follow-up information must be collected<br>• Other, specify:<br>• Our program does not educate our donors on the costs and coverage for OPTN/UNOS mandated post donation living donor routine follow-up |

**Table S2.** Free Text Response Themes

| Policies and procedures for coverage of OPTN/UNOS mandated post donation living donor routine follow-up: Other, specify: | | |
|---|---|---|
| **Theme** | **Number of responses** | **Example text** |
| No written policy | 3 | *We don't have an official policy, but our donors have never encounter problems with this issue and come to us if they have don't have their own insurance.* |
| Guideline or standardized process | 2 | *We have a standard process the financial counselors follow no written policy* |
| Do not know or unsure | 3 | *I do not know* |
| Visits are non-billable and are adjusted off | 1 | *UNOS required post visits are non-billable to donor or recipient's insurance. Institutional adjustment.* |

* 1 response characterized 2 themes

| Mechanisms used for recovering or covering the costs of OPTN/UNOS mandated post donation living donor routine follow-up. Other, specify: | | |
|---|---|---|
| **Theme** | **Number of responses** | **Example text** |
| Write off charges | 4 | *Recipient's ins for the 1st post-donation visit, written off as cost of doing business for 6 mos, 1yr and 2 yr* |
| Reiteration of CMS regulations re: not allowable on cost report | 2 | *Per CMS, those costs are the Tx program's financial responsibility & not allowable to the cost report. PRM was updated in '16 to reflect this.* |
| Bill recipient's insurance and donor insurance | 2 | *If the patient comes here we bill the recipient insurance but if they do it locally the donor's insurance is billed.* |
| Recommend outside support | 1 | *Advise donors to apply for NLDAC support* |
| Use other funds | 1 | *Local funds* |
| Other | 1 | *Possibly other not sure* |

| Timing on donor candidate education on the costs and coverage for OPTN/UNOS mandated post donation living donor routine follow-up. Other, specify: | | |
|---|---|---|
| **Theme** | **Number of responses** | **Example text** |
| Follow up visits | 2 | *At time of follow-up visit* |
| First call with donor prior to donation | 1 | *First call with the donor in regard to donation* |
| Do not educate donors | 1 | *We don't 'educate' donors on these costs because we would never expect them to pay. We would write off these costs if there is no way to bill insurance.* |
| Do not know | 1 | *I do not know* |
